# Dopant-Free and Self-Charged Gel-Type Polyelectrolytes for Supercapacitors

**DOI:** 10.1021/acsomega.5c10696

**Published:** 2026-03-17

**Authors:** Bryan A. Corzo, Hugo Hernández-Martínez, José A. Ávila-Niño, Paola G. Vilchis-Gutiérrez, M. Dolores Durán-García, Minerva Valencia-Ortega, Estrella Ramos, Lilian I. Olvera

**Affiliations:** 1 Instituto de Investigaciones en Materiales, Universidad Nacional Autónoma de México, Apartado postal 70-360, CU, Coyoacán Ciudad de México 04510, México; 2 Secihti - CIATEQ Unidad Jalisco, Nodo Servidor Público 165, Anexo Club de Golf Las Lomas, Zapopan, Jalisco 45136, México; 3 Departamento de Ingeniería Mecánica, Facultad de Ingeniería, UAEMéx, Cerro de Coatepec s/n, Toluca, Estado de México 50110, México

## Abstract

With the increasing demand for wearable and flexible energy storage devices, there has been significant interest in developing safe and mechanically stable prototypes. For this, liquid electrolytes for batteries and supercapacitors (SC) need to be replaced by self-charged gel-type polyelectrolytes (SCGPE), which exhibit good ionic mobility and offer the advantage of being incorporated into solid-state electronic devices. Here, the SCGPE studied has been synthesized by a polyhydroxyalkylation reaction in a superacid medium of 4-acetylpyridine and the nonactivated aromatic compounds *para*-terphenyl and biphenyl. The reaction was carried out in a single step at room temperature, without the use of metal catalysts, and yielded water as the only byproduct. Chemical modification reactions were then carried out by quaternizing 4-acetylpyridine using a bromohexyltrimethylammonium salt, which incorporated positively charged elements onto the polymer backbone. The functionalization degree and viscosity of the gel polyelectrolyte were the two main factors affecting SC performance. These SCGPEs exhibit ionic conductivity without the need for doping with a conducting salt, ionic liquid, or acid, thereby ensuring that the PE maintains its mechanical stability and safety. Molecular dynamics simulations have confirmed the key role of the solvent in influencing the polymer conformation and ion transport. SCGPEs enable the sparing of dopants, such as ionic liquids, conducting salts, or acids, as the SCGPE exhibits good ionic conductivity (on the order of 10^–4^ S/cm) and high specific capacitance (up to 123 mF cm^–2^) when used in textile carbon-based SCs. It also showed only slight differences with a well-known gel polyelectrolyte (GPE), poly­(vinyl alcohol)-potassium hydroxide (PVA-KOH). GPEs used with textile carbon electrodes pave the way for developing all-solid-state wearable SCs without leaking or spilling of liquid electrolytes.

## Graphical abstract

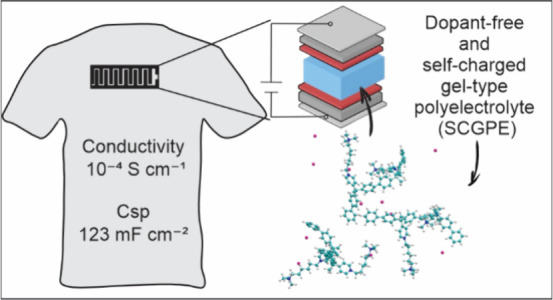

## 1 Introduction

Flexible energy storage devices have attracted significant attention in recent years due to the growing demand for incorporating batteries or supercapacitors (SCs) in flexible electronics environments for portable and wearable applications,^1,2^ with their main advantages being flexibility and lightweightness.^3^ SCs are one of the most developed energy storage devices due to their fast energy delivery, high power density, and high cyclic stability compared with batteries.^4^ Flexible SCs have been developed lately, using flexible substrates like paper,^5^ plastic,^6^ or cotton,^7^ with the latter being the most widely used due to the rise of textile energy devices manufacturing for future flexible electronics. For SC using cotton substrates, carbon-based electrodes are the most commonly used, as they can be easily incorporated into cotton by dipping in a carbon ink^8^ and carbonization,^9^ and are utilized for their high surface area and high conductivity.^10^

In an SC with liquid electrolytes, the mobile solvated anions and cations migrate to the positive and negative electrodes, respectively, due to the application of a potential across the electrodes. This forms an electric double-layer capacitance (EDLC) in the electrode’s interfaces,^11^ where a charge is stored by electrostatic forces.^12^

The development of flexible electrodes for SC has led to finding solutions for developing all-solid-state devices by replacing liquid electrolytes with gel polymer electrolytes (GPEs), which can be incorporated into portable and wearable applications.^13^

The charging phenomenon in GPEs is similar to that observed with liquid electrolytes,^14^ as most gel-type composite electrolytes used in SCs consist of a matrix polymer and additives that carry mobile ions through the gel and form EDLC layers on the surfaces where ionic adsorption–desorption processes occur during SC charging and discharging, respectively.^15^ The additives used to embed mobile ions into the gel-type polymer generally consist of salts, acids, or ionic liquids.^16–18^

GPEs are used to develop solid-state and flexible energy storage devices, such as SCs^11^ and batteries,^19^ with their main advantages being the avoidance of liquid spills and their flexibility.^20^ GPEs must have high ionic mobility through the gel to form EDLC sites and generally lie in the order of 10^–4^ to 10^–2^ S cm^–1^ at room conditions, depending on the additive concentration^21^ and the polymer viscosity^21,^ which lies between viscosities of liquid and solid electrolytes, reducing the leakage of liquid electrolytes, which improves the device’s safety, and reduce the interface and bulk resistance of the solid electrolytes.^19^

Self-charged gel-type polyelectrolytes (SCGPEs) are electrolytes in which the ionic groups are covalently attached to the polymer backbone^23^ and, upon dissociation, generate charged polymer chains along with mobile counterions of opposite charge. When employed in energy storage devices, such as supercapacitors (SCs), the mobile counterions migrate toward the oppositely polarized electrode, while the charged polymer backbone remains immobilized and can contribute to the formation of an electrochemical double-layer capacitance (EDLC) at the other electrode through chain alignment.^11^

The use of SCGPEs eliminates the need for external dopants—such as ionic gels, inorganic salts, or acidic solutions—thereby providing high ionic conductivity arising from the mobility of intrinsic counterions.^23^ Although conventional gel polymer electrolytes (GPEs) are generally easier to prepare and can exhibit relatively high ionic conductivities when doped with external salts (e.g., KOH, LiClO_4_, or NaCl), SCGPEs offer a distinct set of advantages stemming from their intrinsically charged polymeric backbone, which fundamentally differentiates them from classical GPEs. In SCGPEs, the ionic groups are covalently tethered to the polymer chains rather than introduced as external ionic species or liquid plasticizers. As a result, the charge carriers are an integral part of the material, generating a stable and permanent internal ionic environment that is not susceptible to dopant leaching, evaporation, or redistribution during the operation. Furthermore, many dopants commonly employed in GPEs—such as small inorganic salts, ionic liquids, or metal-based species—may be toxic, bioaccumulative, corrosive, or environmentally persistent. Finally, the density of charged monomer units in SCGPEs can be precisely tuned, allowing for control over ionic mobility and electrochemical double-layer capacitance.

In this work, we developed carbon-based SCs in cotton substrates using SCGPEs synthesized via a polyhydroxyalkylation reaction in a superacid medium of 4-acetylpyridine with the nonactivated aromatic compounds *para*-terphenyl and biphenyl. The reaction was carried out in a single step at room temperature, without the use of metal catalysts, and produced water as the only byproduct. Chemical modification reactions were then carried out by quaternization of 4-acetylpyridine using (6-bromohexyl)­trimethylammonium bromide, which incorporated positively charged chains onto the polymer backbone.^24^ Three kinds of aryl-ether-free poly­(arylene pyridine)­s were synthesized by varying the proportion of the two aromatic compounds and used as Random-type copolymers. The use of polymers based on 4-acetylpyridine and *para*-terphenyl, as well as 4-acetylpyridine and biphenyl, for solid polyelectrolytes in anion exchange membranes for high-temperature proton fuel cells has recently been reported. High conductivities were observed for the polymers obtained with biphenyl; however, they exhibited low mechanical properties due to their high swelling capacity and solubility.^25^ For this reason, to overcome this limitation and simultaneously achieve adequate conductivity while preserving mechanical stability, the synthesis of 4-acetylpyridine, biphenyl, and *para*-terphenyl copolymers was proposed (1B_*x*_T_*y*_).

SCs developed in this work consist of two textile electrodes impregnated with carbon ink and a cellulose separator impregnated with the gel polyelectrolytes; meanwhile, electrochemical characterizations were performed to obtain the specific capacitance, power, and energy densities.

## 2 Experimental Part

### 2.1 Materials

All raw materials were obtained from Sigma-Aldrich. Solvents and catalyst compounds, such as dichloromethane (CH_2_Cl_2_), *N*-methyl-2-pyrrolidone (NMP), dimethyl sulfoxide (DMSO), trifluoroacetic acid (TFA), trifluoromethanesulfonic acid (TFSA), and 4-acetylpiridine, were distilled before use, and (6-bromohexyl)­trimethylammonium bromide was washed with hexane to remove existing impurities before use. Biphenyl, *p*-terphenyl, phosphoric acid, and 2,2,2-trifluoroethanol (TFE) were used as received. Activated carbon (AC) for Sigma-Aldrich labeled as ″charcoal activated granular, 4–14 mesh″ with a molecular weight of 12.01 g·mol^–1^ was used for electrode fabrication.

### 2.2 Synthesis

The synthesis of copolymers was carried out via a polyhydroxyalkylation reaction in a superacid medium of 4-acetylpyridine with biphenyl and *p*-terphenyl. It has been observed that the use of biphenyl in this type of polymer provides high solubility and good ionic conductivity, while polymers with *p*-terphenyl exhibit decreased conductivity but greater mechanical stability. Therefore, obtaining copolymers by varying the proportions of biphenyl (Bx) and *p*-terphenyl (Ty) (where *x* and *y* are the proportions of biphenyl and *para*-terphenyl fragments) will help achieve a favorable balance between mechanical stability and good ionic conductivity (Figure 1).

**Figure 1:**
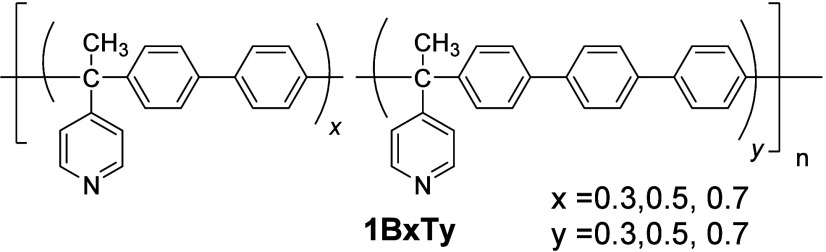
Structure of copolymers.

Copolymer **1B**_**0.3**_ **T**_**0.7**_ was synthesized via a polyhydroxyalkylation reaction in superacid media under nonstoichiometric conditions. A flask equipped with a mechanical stirrer was placed with 4-acetylpiridine (2.3637 g, 19.51 mmol), biphenyl (0.6936 g, 4.50 mmol), *p*-terphenyl (2.4165 g, 10.49 mmol), and dichloromethane (15 mL). The flask was placed into an ice bath, and 0.6 mL of TFA and 15 mL of TFSA were added slowly. The reaction was carried out at room temperature under stirring for 21 h, resulting in a viscous homogeneous dark-blue solution. The homogeneous reaction mixture was slowly poured into an excess of deionized water under vigorous stirring, resulting in the immediate formation of creamy white fibrous precipitates. The fibers were then collected by filtration and washed repeatedly with fresh deionized water. The copolymer was dissolved in NMP and then precipitated in a sodium hydroxide solution for deprotonation, followed by thorough washing with water. Then, the crude copolymer was first dissolved in *N*-methyl-2-pyrrolidone (NMP) to obtain a clear solution. This solution was then slowly poured into an excess of deionized water under stirring, inducing copolymer precipitation to remove residual monomers and low-molecular-weight species. The resulting solid was collected by filtration, washed thoroughly with deionized water, and dried. The resulting pure fibers (4.3945 g, 94% yield) displayed an inherent viscosity of 0.73 dL g^–1^ in the NMP.

Copolymer **1B**_**0.5**_ **T**_**0.5**_ was synthesized in a manner similar to that of copolymer **1B**_**0.3**_ **T**_**0.7**_. 4-Acetylpiridine (2.3774 g, 19.63 mmol), biphenyl (1.1576 g, 7.51 mmol), *p*-terphenyl (1.7276 g, 7.50 mmol), dichloromethane (15 mL), TFA (0.6 mL), and TFSA (15 mL) were stirred at room temperature for 3 days, resulting in a viscous homogeneous dark-blue solution which was subsequently poured into deionized water. Creamy white fibers were obtained and washed intensively with deionized water. The copolymer was first dissolved in NMP, precipitated in a sodium hydroxide solution, and washed with water to achieve deprotonation. Then, the crude copolymer was first dissolved in *N*-methyl-2-pyrrolidone (NMP) to obtain a clear solution. This solution was then slowly poured into an excess of deionized water under stirring, inducing copolymer precipitation to remove residual monomers and low-molecular-weight species. The resulting pure white fibers (4.1081g, 93% yield) displayed an inherent viscosity of 0.85 dL g^–1^ in NMP.

Copolymer 1B_**0.7**_ **T**_**0.3**_: A mixture of 4-acetylpiridine (2.3663 g, 19.53 mmol), biphenyl (1.6198 g, 10.50 mmol), and *p*-terphenyl (1.0407 g, 4.52 mmol), dichloromethane (15 mL), TFA (0.6 mL), and TFSA (15 mL) was stirred at room temperature for 2 days. After this time, a viscous homogeneous dark-blue solution was obtained and was subsequently poured into water. A creamy white fiber color was obtained and washed thoroughly with deionized water. Deprotonation of the copolymer was carried out by dissolving it in NMP, precipitating it in a sodium hydroxide solution, and washing it thoroughly with water. Then, the crude copolymer was first dissolved in *N*-methyl-2-pyrrolidone (NMP) to obtain a clear solution. This solution was then slowly poured into an excess of deionized water under stirring, inducing copolymer precipitation to remove residual monomers and low-molecular-weight species. The resulting pure white fibers (4.0615 g, 91% yield) displayed an inherent viscosity of 0.78 dL g^–1^ in NMP.

#### 2.2.1 Functionalized Copolymers (M1BxTy)

Postsynthesis chemical modification reactions of copolymers were carried out to incorporate a quaternary ammonium group using (6-bromohexyl)­trimethylammonium bromide salt. A 10% (w/v) solution of each copolymer in NMP was prepared. A brominated salt was subsequently added in a 1:3 molar ratio. The mixture was stirred for 5 days at room temperature, followed by precipitation in ethyl acetate. The precipitate was subjected to multiple washes with warm ethyl acetate. The obtained fibers were then dried overnight. The degree of functionalization for each modification was confirmed by a ^1^H NMR analysis. The modified polymers (SCGPE) are named as **M1BxTy** (where *x* and *y* are the proportion of biphenyl and *para*-terphenyl fragments, *x* = 0.3, 0.5, and 0.7 and *y* = 0.3, 0.5, and 0.7)

### 2.3 GPE-Supercapacitor Preparation

Various SCGPEs were prepared by using different methods. For the modified copolymers, gels were formed by dissolving the polyelectrolyte in a 10% w/v solution of DMSO or TFE. For the PVA-KOH GPE, an aqueous 1 M KOH electrolyte was stirred for 24 h at ambient conditions. Then, PVA was added to form a 1:1 (PVA-KOH) solution, and the mixture was further stirred for 3 h.

### 2.4 Carbon Electrode Preparation

The fabrication of the textile-activated carbon–nickel textile electrode was performed by the impregnation of an AC ink on a cotton substrate. The activated carbon was mechanically milled to reduce particle size and obtain a homogeneous, stable ink suitable for impregnating the textile substrate. This milling step is necessary to break down large agglomerates and improve the dispersion of activated carbon in water. The ink was prepared using 1 g of activated carbon dispersed in 50 mL of deionized water, in addition to 50 ML of the surfactant dodecylbenzenesulfonate (DBSS), and then the cotton substrates were immersed in it. Finally, they were dried at 110 °C for 40 min in a hot plate.^8^ The Brunauer–Emmett–Teller (BET) specific surface area of the electrodes was 243.05 m^2^ g^–1^, as measured using an Autosorb iQ instrument.

### 2.5 Fabrication of a Supercapacitor

A test supercapacitor with a two-electrode cell configuration was prepared by using two carbon electrodes, separated by a gel polyelectrolyte and a cellulose separator. Two circular disks of stainless-steel coins, each with a diameter of 2 cm, were used as the current collectors. Textile carbon electrodes were cut into circles with a diameter of 2 cm. A filter separator of mixed cellulose (Millipore filter, 0.45 μm pore size, 150 μm thick) was soaked in gel polyelectrolytes for 30 min before the measurements.^8^

For measurement, we used a two-electrode stainless-steel cell with a mechanical mechanism to press the SC, ensuring low resistance between the current collector and the electrode. In this architecture, an insulating separator is necessary despite the presence of gel polyelectrolytes. The model of the 2-electrode supercapacitor device assembly and photographs of the closed and opened cell are shown in Figure S1.

### 2.6 Characterization

Molecular structures of polymer precursors and cationic materials were determined by NMR spectroscopy on a Bruker Avance 400 spectrometer using chloroform-d (CDCl3) and dimethyl sulfoxide-*d*_6_ (DMSO-*d*) as solvents. A Thermo Scientific Nicolet iS10 FTIR-ATR spectrometer was used to acquire the FTIR spectra of the base polymers and polyelectrolytes. The inherent viscosities were determined using a Ubbelohde viscometer at 25 °C with solutions of 0.2% w/v base polymers and cationic materials in NMP and DMSO, respectively, using eq 1:

$$\eta_{h}=\frac{\ln\left(\frac{t_{1}}{t_{0}}\right)}{C}$$ 1

where *t*_1_ and *t*_0_ are the sample and blank fall times, respectively, and *C* is the solution concentration. Thermogravimetric analyses (TGA) were carried out under nitrogen and air at a heating rate of 10 °C/min using a TA Instruments TGA-Q50.

Cyclic voltammetry (CV), galvanostatic charge–discharge (GCD), and electrochemical impedance spectroscopy (EIS) measurements were performed by using a Biologic SP-150 potentiostat. The EIS measurements of the SCs were conducted over a frequency range of 1 Hz to 100 kHz.

The ionic conductivity of the gel-type polymer was obtained using the eq 2:

$$\sigma =\frac{L}{R_{s}\times A}$$ 2

where *R_s_* is the solution resistance of the gel polymer obtained from the intercept with the real axis at high frequencies in the Nyquist plot, *L* is the distance separation between electrodes, and *A* is the area of the electrodes.

The CV measurements were carried out in a potential window of −1 to 1 V at various scan rates, from 10 to 100 mV/s. The GCD test was performed over a range of voltages from −1 to 1 V at current densities of 3, 4, 5, and 6 mA/cm^2^. The areal capacitance of the SCs was obtained from the discharge profile using eq 3:

$$C_{\mathrm{GCD}}=\frac{2i\int Udt}{\Delta U^{2\times }A}$$ 3

where *i* is the discharge current rate, ∫*Udt* represents the discharge profile area, Δ*U* is the potential difference of the discharge profile, excluding the initial potential drop of the discharge curve, and *A* is the area of the electrodes. The energy (*E*) and power (*P*) densities were calculated by the following equations:

$$E=\frac{1}{2}C_{\mathrm{GCD}}{\left(△V\right)}^{2}$$ 4

$$P=\frac{E}{\Delta t}$$ 5

### 2.7 Molecular Dynamics

#### 2.7.1 System Setup

Molecular dynamics (MD) simulations were performed using the GROMACS 2023.5 package^26^ employing the GROMOS54A7 ^27,28^ force field. The model system consisted of three GPE polymer fragments solvated either in DMSO or TFE. Each polymer fragment comprised three monomer units based on the 1 acetylpyridine and B biphenyl (acetylpyridine–biphenyl) repeating unit, functionalized with hexadecyltrimethylammonium groups to form the cationic polymeric fragment. The system was electrically neutralized by the addition of bromide counterions (Br^–^). The systems were placed in cubic simulation boxes of 4 × 4 × 4 nm^3^ with periodic boundary conditions in all directions.

#### 2.7.2 Simulation Protocol

Initial simulations of the pure solvents were conducted to verify that their computed densities matched the experimental values. Subsequently, the cationic polymeric fragments and counterions were inserted into the solvent box that had been equilibrated. All systems underwent energy minimization using the steepest descent algorithm^29^ to remove steric clashes and correct unfavorable geometries. Production simulations were performed in the NPT ensemble for 50 ns at 293.15 K and 1 bar. Electrostatic interactions were computed using the Particle Mesh Ewald (PME) method^30^ with a cutoff of 1.2 nm for both Coulomb and van der Waals interactions. A static electric field of 1 V was applied to the system to analyze the effect on the polymer chain arrangement.

#### 2.7.3 Structural Analysis

To characterize the spatial organization, mass density profiles along the *z*-axis (ρ_*n*_(*z*)) were computed for both the cationic polymeric fragment and Br^–^ counterions. Densities were averaged over the final 50 ns of the trajectories. The mass density profile is represented in eq 6.

$$\rho_{n}\left(z\right)=\frac{\varphi }{NL_{x}L_{y}\Delta z}\sum_{i=1}^{N}n_{i}\left(z\right)$$ 6

where *N* is the total number of configurations, *n*_*i*_(*z*) represents the number of atoms or molecules within each slice along *z*, *L_*x*_* and *L_*y*_* are the dimensions of the simulation box in the respective directions, and φ is a conversion factor from number density to mass density, using Avogadro’s number and the molecular mass of the species.

Additionally, radial distribution functions (*g*_*AB*_(*r*)) were calculated to analyze the local structure surrounding the cationic polymeric fragment. This pair distribution function indicates the probability of finding a particle at a distance of r from a reference particle. In this case, *g*_*AB*_(*r*) describes the probability of finding a Br^–^ ion at a distance *r* from a cationic polymeric fragment, and is given by eq 7:

$$g_{AB}\left(r\right)=\frac{V\sum_{i\in A}^{N_{A}}\sum_{j\in B}^{N_{B}}P\left(r\right)}{4\pi r^{2}}$$ 7

where *V* is the system volume, *P*(*r*) is the probability of finding a Br^–^ ion at a distance *r* from the cationic polymeric fragment, and the denominator accounts for the volume of a spherical shell at that distance.

#### 2.7.4 Simulation under Electric Field

To evaluate the response of the system to an external field, additional simulations were performed with a static electric field of 1 V applied along the +*x* direction. The field remained active during the entire production phase. Structural changes were analyzed in terms of polymer fragment alignment and the redistribution of counterions.

## 3 Results and Discussion

### 3.1 Polymer Synthesis

The precursor copolymers were obtained via a superacid polyhydroxyalkylation reaction using TFSA and a mixture of TFA and CH_2_Cl_2_ at room temperature (Scheme 1). This polymerization method enables the synthesis of high-molecular-weight polymers without ether bonds in the polymer backbone, resulting in materials with exceptional thermal, mechanical, and chemical stability. The main criteria for monomer selection were based on studies conducted by Cetina-Macilla et al.^24^ and Y. Jin et al.,^25^ which confirmed that resulting polymers exhibited high solubility in various organic solvents.

**Scheme 1:**
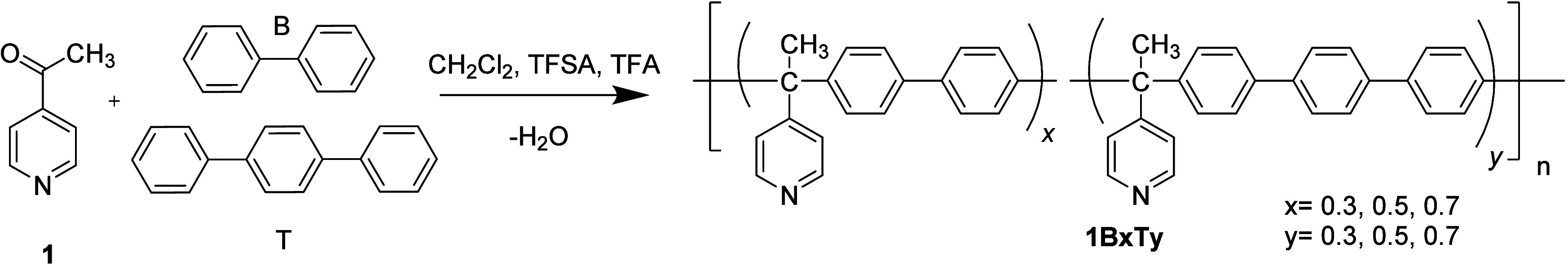
Copolymers, Polymerization of 4-Acetylpiridine with Biphenyl and *p*-Terphenyl

Additionally, incorporating a heterocyclic ring enabled further modification reactions, making these functional materials highly attractive for a wide range of applications.

The SCGPEs were synthesized by an alkylation reaction on the pyridine fragment using (6-bromohexyl)­trimethylammonium bromide (Scheme 2), incorporating positively charged chains onto the polymer backbone. The results of the modifications are depicted in Table 1. The modification was confirmed by FTIR and ^1^H NMR spectra.

**Scheme 2:**
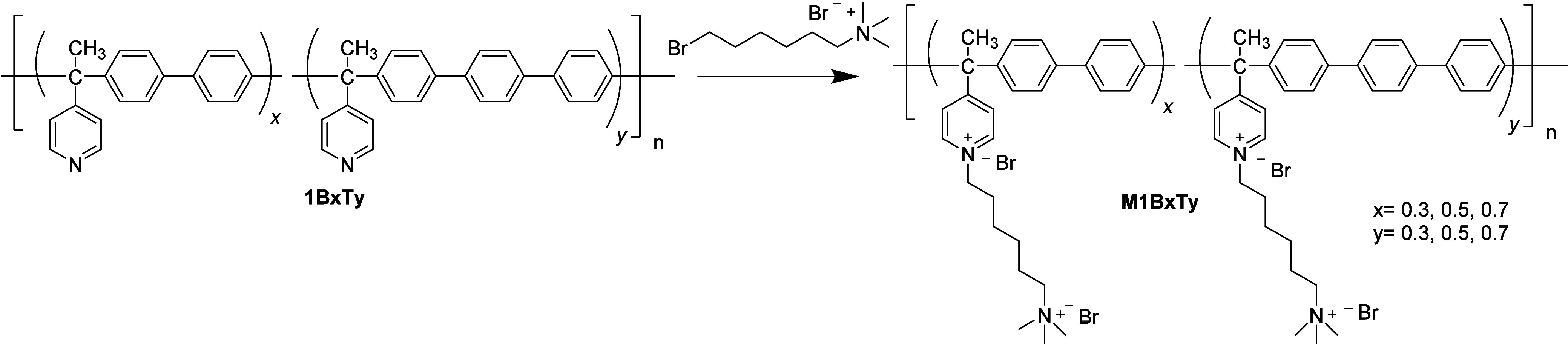
Synthesis for Solid SCGPEs **M1B**_**0.3**_ **T**_**0.7**_, **M1B**_**0.5**_ **T** **0.5**, and **M1B**_**0.7**_ **T**_**0.3**_

**Table 1:** Modifications Results for Cationic Polymers

ID	base polymer (mmol)	modification reagent (mmol)	reaction time	functionalization %	inherent viscosity (dL g^–1^)
M1B_0_._3_T_0_._70_	3.0190	9.0571	5 days	86	3.25
M1B_0_._5_T_0_._5_	3.2080	9.1391	5 days	100	2.68
M1B_0_._7_T_0_._3_	2.9982	9.2975	5 days	91	3.03

### 3.2 Polymer Characterizations

FTIR spectra of polymer **1B**_**0.5**_ **T**_**0.5**_ and SCGPE **M1B**_**0.5**_ **T**_**0.5**_are shown in Figure 2. If we compare the two spectra, in the range of 2950 to 3000 cm^–1^, the characteristic signals of the aromatic and aliphatic C–H vibrations are observed in the **1B**_**0.5**_ **T**_**0.5**_ spectrum. In this region, the polyelectrolytes (quaternary salts) also exhibit a signal around 3400 cm^–1^, characteristic of the O–H stretching of water. This feature arises due to the hygroscopic nature of the resulting polyelectrolyte, which contains cationic groups and readily absorb atmospheric moisture. The bands at 1600–1480 cm^–1^ are attributed to ring skeletal vibrations (C=C/C=N), while the C–N stretching vibrations are assigned to the band observed around 1350 cm^–1^. The polyelectrolytes exhibit a strong band at 1640 cm^–1^, which is not present in the pyridine polymer spectrum. This band has been assigned to C=N vibrations characteristic of the quaternary nitrogen atom in a heterocyclic ring (assigned to ring vibrations of the pyridinium ion). The band at 1210 cm^–1^ is assigned to aromatic ring skeletal vibrations (coupled C=C/C=N modes) of the pyridinium moiety, while the band at 1080 cm^–1^ is attributed to C–N stretching vibrations of the alkyl side chain. Sharp bands at 1001 and 798 cm^–1^ can be representative of the vibrational deformations of aromatic C–H groups.

**Figure 2:**
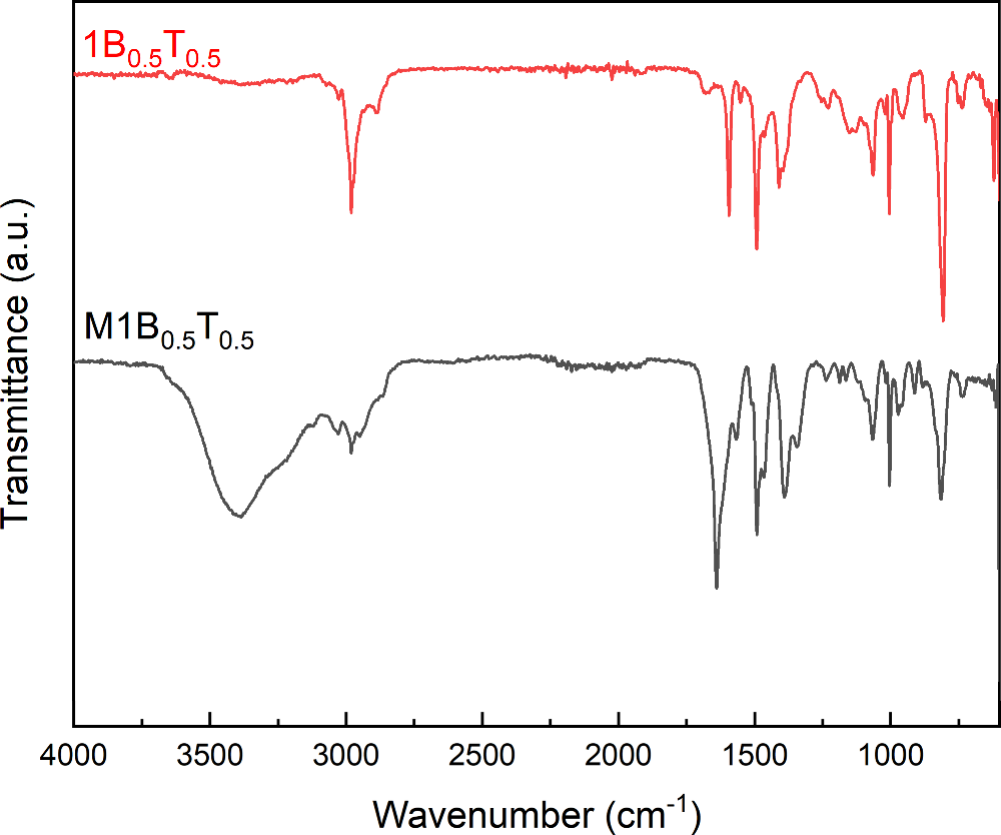
FT-IR spectra for the base copolymer (**1B**_**0.5**_ **T**_**0.5**_) and polyelectrolyte (**M1B**_**0.5**_ **T**_**0.5**_).

Figure 3 shows the NMR spectrum of polymer **1B**_**0.5**_ **T**_**0.5**_ and polyelectrolyte M1B_50_T_50_. The presence of new signals in the 1.3 to 5.0 ppm range in polyelectrolyte **M1B**_**0.5**_ **T**_**0.5**_corresponds to the aliphatic protons belonging to (6-bromohexyl)­trimethylammonium bromide grafted. This confirms the incorporation of the cationic group into the main chain. The pyridine 8H signal shifts from 8.5 to 9.2 ppm in polyelectrolytes associated with the presence of the pyridinium group; the complete disappearance of the signal at 8.5 ppm would indicate total modification, i.e., a 100% functionalization degree. In cases with lower functionalization degrees, the unmodified residue could be observed as a small signal around 8.5 ppm. The degree of modification for the obtained polyelectrolytes was 86, 100, and 91% for **M1B**_**0.3**_ **T**_**0.7**_, **M1B**_**0.5**_ **T**_**0.5**_, and **M1B**_**0.7**_ **T**_**0.3**_, respectively (Figures S2 and S3).

**Figure 3:**
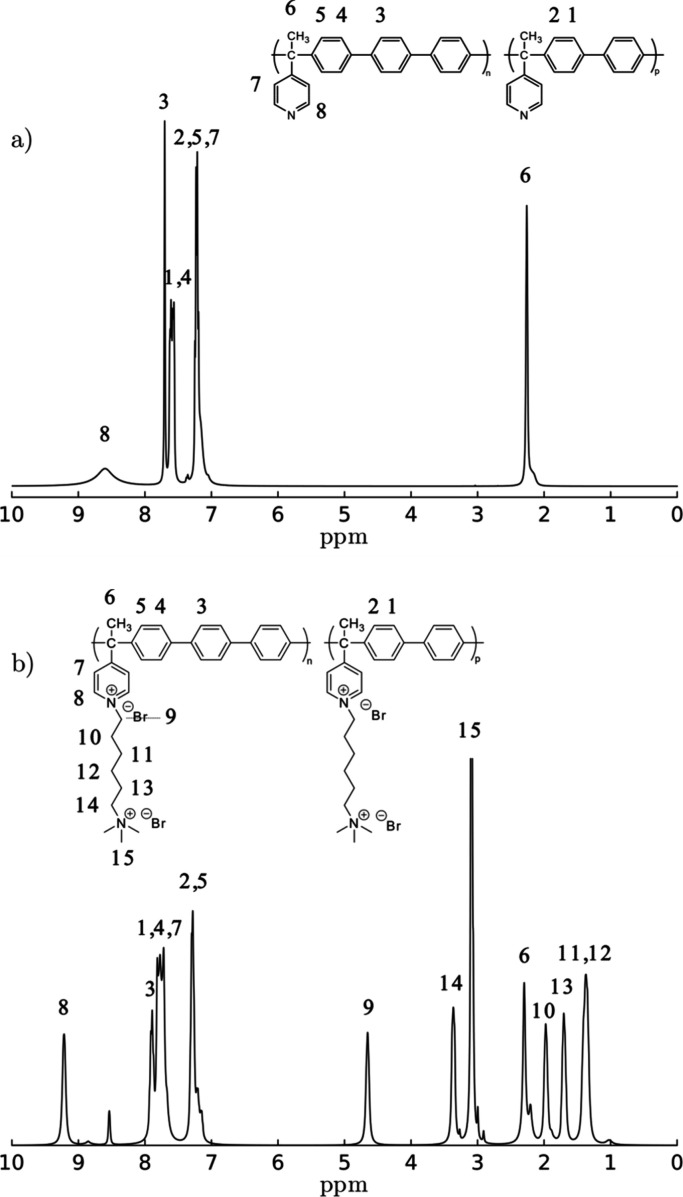
^1^H NMR spectra of the base copolymer **1B**_**0.5**_ **T**_**0.5**_ (a) and polyelectrolyte **M1B**_**0.5**_ **T**_**0.5**_(b).

It is widely recognized that electrophilic substitution reactions on aromatic systems, such as alkylation and acylation, typically produce mixtures of isomers. Additionally, it is generally expected that the ^1^H NMR spectra of large, fully aromatic polymers would exhibit significant broadening. However, what is particularly noteworthy in the presented polymers and polyelectrolytes is that the NMR spectra display sharp, well-defined signals with all expected resonances clearly observable. This indicates a high degree of regioselectivity in the polymerization process, which is facilitated by superacid-catalyzed polyhydroxyalkylation.

### 3.3 Polymer Properties

The obtained materials appear as white and slightly yellowish fibers for pyridine copolymers and polyelectrolytes, respectively. Unmodified polymers are soluble in most organic solvents, including low-polarity chlorinated solvents, whereas polyelectrolytes are soluble in polar solvents such as DMSO, DMF, and TFE, among others, as seen in Table 2. This characteristic facilitates their processing through various casting techniques to produce transparent, mechanically robust, and flexible films. Figure 4 illustrates the thermal stabilities of **1B**_**0.5**_ **T**_**0.5**_ and **M1B**_**0.5**_ **T**_**0.5**_, as determined through TGA analysis in air and nitrogen atmospheres. The unmodified polymer **1B**_**0.5**_ **T**_**0.5**_ exhibited a single decomposition stage at around 550 °C, corresponding to its degradation and indicating high thermal stability. A similar pattern was observed for **1B**_**0.3**_ **T**_**0.7**_ and **1B**_**0.7**_ **T**_**0.3**_ Figure S4. The polyelectrolyte **M1B**_**0.5**_ **T**_**0.5**_ showed a two-step thermal decomposition pattern. The loss of weight in the first step, from 210 to 224 °C, is presumably due to decomposition of the side chain and the cationic groups. The second step, above 400 °C, could be ascribed to the decomposition of the polymer backbone. A similar behavior is observed for the other polyelectrolytes.

**Figure 4:**
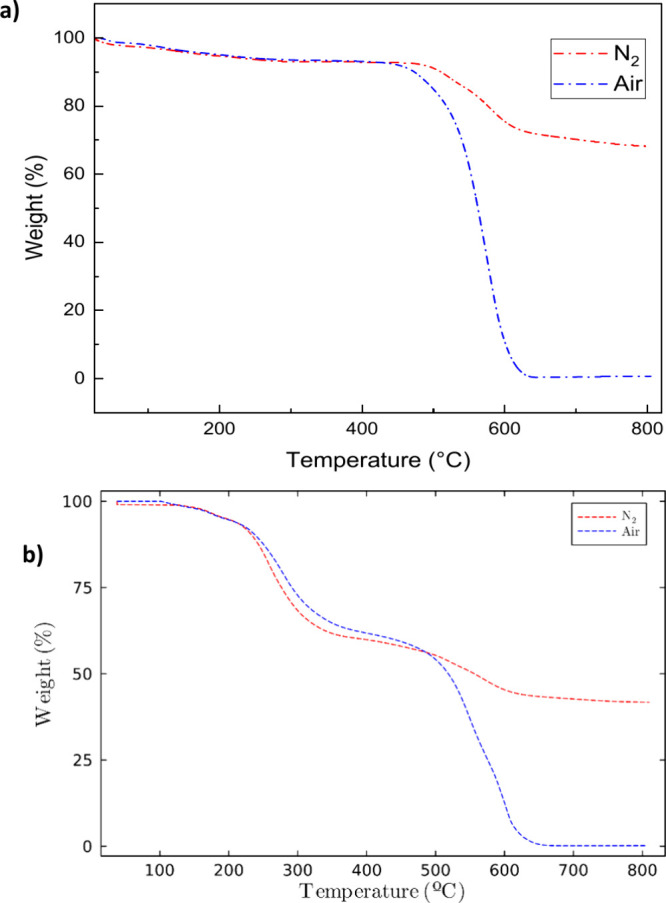
TGA curves of (a) **1B**_**0.5**_ **T**_**0.5**_ and (b) **M1B**_**0.5**_ **T**_**0.5**_ under a N_2_ and air atmosphere.

**Table 2:** Modifications Results for Cationic Polymers

	Td (°C)		char yield, 800 °C (%)	solvent							
ID	N_2_	Air	N_2_	CHCl_3_	DMF	DMAAc	DMSO	THF	NMP	Acetone	TFE
1B_0.3_T_0.7_	493	474	66	+	–	–	–	+	+	+	+
1B_0.5_T_0.5_	494	488	68	+	–	–	–	+	+	+	+
1B_0.7_T_0.3_	497	508	69	+	–	–	–	+	+	+	+
M1B_0.3_T_0.7_	497	484	34	–	+	+	+	–	+	–	+
M1B_0.5_T_0.5_	504	527	42	–	+	+	+	–	+	–	+
M1B_0.7_T_0.3_	495	560	40	–	+	+	+	–	+	–	+

The SCGPEs were prepared in two polar solvents with different boiling points and viscosities, which facilitated their ease of handling. However, differences in behavior were observed.

### 3.4 Solvent Effect

Different organogel polymers were obtained by dissolving a 10% (w/v) cationic fiber solution in DMSO or TFE. In SCGPEs for use in SCs, various factors, such as solution concentration, solvent type, and interactions between the polymer and other components, are critical to performance. Selecting an appropriate electrolyte requires considering various criteria, such as low viscosity, high ionic conductivity, and a wide electrochemical potential window, among others.

The SCGPEs were soluble in different solvents such as DMSO and TFE, with TFE being one of the most promising solvents as it could dissolve all the synthesized polyelectrolytes, which means that TFE allows a clear separation between charges from the polymer backbone and the counterions when an electric field is applied, which was proved by molecular dynamics simulation. This increases the ion mobility, leading to the formation of more EDLC sites at the electrolyte/electrode interfaces. In addition, the SCGPEs showed a better elongation of the charged chains in the direction of the electric field in TFE compared to another solvent (DMSO), allowing the counterions Br^–^ to migrate to the opposite electrode.

MD simulations revealed a more favorable charge distribution in the case of SCGPEs in TFE, indicating enhanced ion mobility at the electrode interface and resulting in a more compact electric double layer. When an external potential was applied, the MD simulations revealed an alignment of the polymer’s cationic terminals in the direction of the field for the SCGPEs in TFE. A different behavior was observed for the SCGPEs in DMSO, where the polymer chain compacted upon the application of an external field.

### 3.5 Molecular Dynamics

A comprehensive comparison of the behavior of polymer fragments in DMSO and TFE is illustrated in Figure 4, which includes representative conformations, spatial organization, radial distribution functions, and their structural responses to an applied electric field.

#### 3.5.1 Polymer Conformation

Representative snapshots of the polymer fragments in DMSO and TFE reveal marked differences in their conformational behavior. In DMSO, the polymer fragments adopt compact, folded structures that are characterized by significant intramolecular packing. This conformation limits the exposure of charged groups, potentially restricting the accessibility and mobility of the counterions. In contrast, in TFE, polymer fragments appear more extended and relaxed with reduced folding and increased spatial separation. This open configuration facilitates access to the charged sites, likely promoting counterion redistribution and contributing to enhanced ionic mobility.

#### 3.5.2 Density Profiles

Figure 5 presents the mass density profiles along the *z*-axis for the cationic polymeric fragment and Br^–^ ions in DMSO and TFE. In DMSO, the polymeric fragments exhibit a single, broad maximum spanning approximately 0.3–4.0 nm, indicative of a compact polymer conformation. The Br^–^ distribution is relatively uniform and overlaps with the cationic polymeric fragment peak, suggesting strong ion pairing.

**Figure 5:**
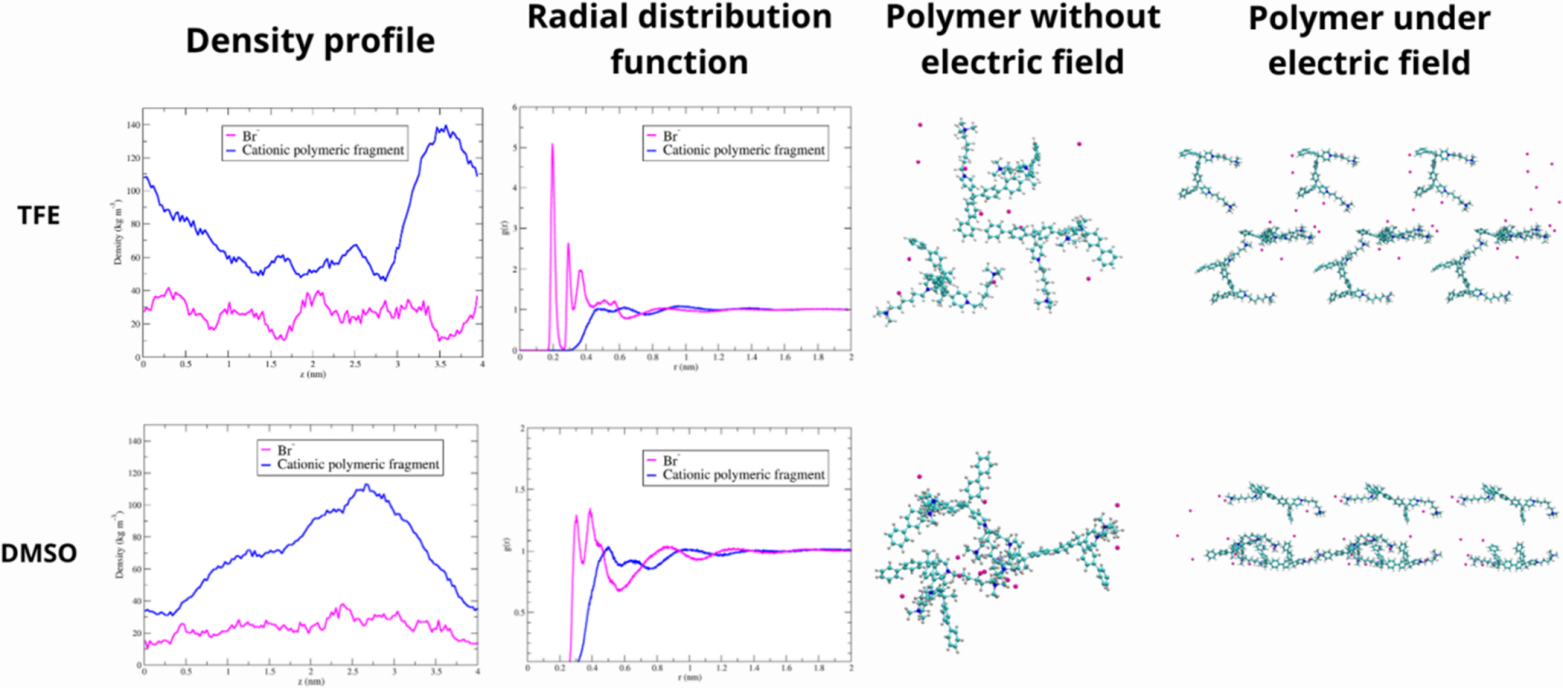
Structural organization of cationic polymeric fragments (blue) and Br^–^ ions (magenta) in TFE (top) and DMSO (bottom). Columns show (1) mass density profiles, (2) radial distribution functions, (3) representative conformations without an electric field, and (4) final configurations under a 1 V electric field. TFE promotes extended polymer conformations, broader Br^–^ ion distribution, and an enhanced response to the applied field compared to DMSO.

In contrast, in TFE the polymeric fragments display a distribution characterized by three distinct regions. Avoiding the density enhancement at *z* ≈ 0 nm associated with periodic boundary effects, the first maximum appears between 1.4 and 1.8 nm, the second between 2.3 and 2.8 nm, and the third between 3.2 and 3.6 nm. This multimodal profile is consistent with a more extended and conformationally heterogeneous polymer structure. Although the overall Br^–^ distribution remains relatively uniform, pronounced spatial fluctuations are observed. Specifically, an increase in the Br^–^ density between 1.6 and 2.4 nm coincides with a region of reduced polymer density, while a second Br^–^ enrichment between 0.8 and 1.6 nm corresponds to another local minimum in the polymeric fragment distribution.

These observations indicate a partial spatial decoupling between Br^–^ ions and the cationic polymeric fragments in TFE compared to DMSO. Such decoupling is consistent with enhanced counterion mobility and, consequently, improved ionic conductivity in TFE.

#### 3.5.3 Radial Distribution Functions

In DMSO, the Br^–^ *g*_*AB*_(*r*) shows a coordination region at short distance followed by a gradual decay, indicating short-range association with the polymeric fragments without pronounced structural ordering. The interaction distances are slightly longer than those in TFE, possibly due to solvation effects or limited access to charged sites in the compact conformation. In TFE, the Br^–^ *g*_*AB*_(*r*) exhibits a sharper and more intense first maximum at shorter distances, accompanied by modulations. This pattern suggests a more defined local arrangement of Br^–^ around the polymer, likely facilitated by the extended chain conformation and increased level of exposure of charged groups. The *g*_*AB*_(*r*) for the cation polymeric fragments remains relatively flat in both solvents, consistent with the spatial separation observed in the density profiles. These differences in local organization support the notion that TFE enables closer ion–polymer contact and greater counterion mobility, in contrast to the more constrained environment in DMSO.

#### 3.5.4 Electric Field Effects

To evaluate how the solvent environment influences polymer behavior under an external field, additional simulations were carried out with a uniform electric field applied along the +*x* direction.

In DMSO, cationic polymeric fragments exhibit partial alignment with the field but remain predominantly folded or compact, consistent with the conformations observed earlier. This limited structural reorganization suggests that the polymer conformation is strongly stabilized by intramolecular interactions or solvation effects, which hinder full extension. The Br^–^ ions remain closely associated with the cationic polymeric fragments, showing little spatial redistribution in response to the field, which indicates limited charge separation and restricted ion mobility. In contrast, in TFE, cationic polymeric fragments adopt more extended conformations, with several aligning along the direction of the applied field. This structural reorganization facilitates greater exposure of charged sites, enabling a partial reorientation and redistribution of the counterions. The Br^–^ ions appear more dispersed, with many clearly located away from the polymer backbone, consistent with increased ion dissociation. Figure S5 illustrates the changes induced by the electric field in both the density profiles and the radial distribution functions. These observations suggest a greater capacity for directional ion transport in TFE, aligning with its higher experimentally observed conductivity.

### 3.6 Ion Conductivity and Supercapacitor Performance

Polymer/solvent interactions directly influence the physicochemical properties of the SCs. The supramolecular arrangement between the polymer chains and the charge distribution in the solvent affect the formation of the electric double layer and, consequently, the capacitance exhibited by the SC. A clear separation between the charges on the polymer chain and the counterions leads to greater mobility and alignment of ions at the electrode interface, thereby compacting the electric double layer and increasing the capacitance. Figure 6 shows the voltammograms obtained for the M1B_50_T_50_ sample in DMSO and TFE. It can be observed that the capacitance for the SCGPEs in DMSO has a lower current than that obtained in TFE. Additionally, the SCGPEs with TFE solvent exhibit a more rectangular behavior compared with the SC with DMSO, indicating a higher charge storage capacity. This behavior is also observed for GPE **M1B**_**0.3**_ **T**_**0.7**_ and **M1B**_**0.7**_ **T**_**0.3**_ (not shown).

**Figure 6:**
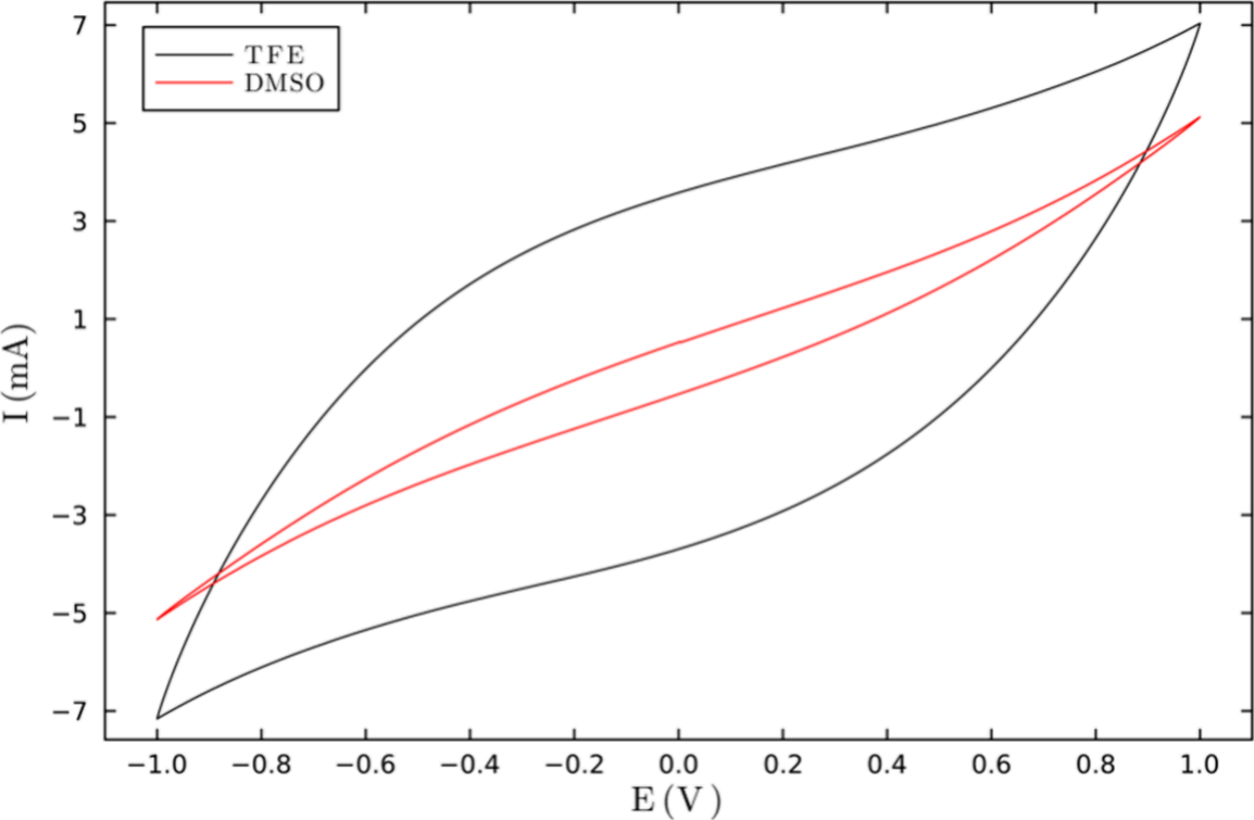
Voltammogram of SC′s for GPE for **M1B**_**0.5**_ **T**_**0.5**_ at a scan rate of 10 mV/s.

Although the polymers are soluble in *N*-methyl-2-pyrrolidone (NMP), this solvent was not selected for electrochemical studies. TFE and DMSO were intentionally chosen because their physicochemical properties strongly influence polymer–solvent interactions and ion dissociation behavior. TFE, as a highly polar protic solvent with strong hydrogen-bonding capability, promotes the effective separation of pyridinium charges from their counterions, leading to enhanced ionic mobility and a more compact electric double layer at the electrode interface. DMSO, a polar aprotic solvent with a high dielectric constant, was used for comparison to evaluate the effect of solvent–polymer supramolecular interactions on the electrochemical response. In contrast, NMP exhibits stronger specific interactions with the polymer backbone and higher viscosity, which hinder efficient ion mobility and make it less suitable for SCGPE electrochemical characterization

The ionic conductivity of different gel polymer electrolytes in TFE was measured by EIS following eq 2. The solution resistance (*R*_s_), which in this context refers to the GPE resistance, was determined by the intersection with the real axis at high frequencies on the Nyquist plots (Figure S6). To maintain a consistent comparison across the synthesized SCGPEs, a GPE used as a reference was established using PVA-KOH at a concentration of 10% w/v. The values of the conductivity are summarized in Table 3, where it is observed that the most conductive gel polyelectrolyte is PVA-KOH, due to the presence of dissociated mobile ions (7.52 × 10^– 4^ S/cm).

**Table 3:** Values of Conductivity, Capacitance, Energy Density, and Power Density for TFE-SCGPEs and PVA-KOH GPE

polyelectrolyte	^c^σ (S cm^–1^)	^a^ *C*_VC_ (mF cm^–2^)	^b^ *C*_GCD_ (mF cm^–2^)	^b^ *E* (μW h cm^–2^)	^b^ *P* (mW cm^–2^)
M1B_0.3_T_0.7_	1.61 × 10^–4^	43.80	68.16	20.23	0.57
M1B_0.5_T_0.5_	2.53 × 10^–4^	84.71	123.15	49.37	0.52
M1B_0.7_T_0.3_	2.46 × 10^–4^	46.43	89.36	29.09	0.57
PVA-KOH	7.52 × 10^–4^	101.82	127.89	60.10	0.51

a Areal capacitance obtained by CV at 10 mV/s.

b Results obtained by GCD profiles at 3 mA cm^–2^

c Ion conductivity obtained from Nyquist plots fitted with Zview.

The solvent–polymer interaction plays a crucial role in determining the physical properties of the SCGPEs. TFE, being a protic solvent, facilitates hydrogen bonding with the charged segments of the polymer chain, significantly reducing solution viscosity and enhancing ionic conductivity. Increasing the ionic conductivity suggests an increase in the double-layer capacitance. As capacitance in EDCL is highly dependent on the electrode porosity. The electrochemical properties and performance of the SCs were evaluated using cyclic voltammetry (CV) in a potential window of −1 to 1 V at different scan rates (Figure 7). As can be observed, Figure 7b,d, which represent **M1B**_**0.5**_ **T**_**0.5**_ SCGPEs and PVA-KHO GPEs, respectively, present higher current values and higher capacitance, which are proportional to their charge storage ability. PVA-KOH GPE is a well-known electrolyte in SCs and is chosen for its excellent ionic mobility due to the high dissociation of K^+^ and OH^–^ ions in the PVA matrix. Then, the similar voltammogram obtained with the **M1B**_**0.5**_ **T**_**0.5**_ SCGPE indicates that this synthesized GPE is a dopant-free option for use in SCs, enhancing the ionic mobility and charge storage.

**Figure 7:**
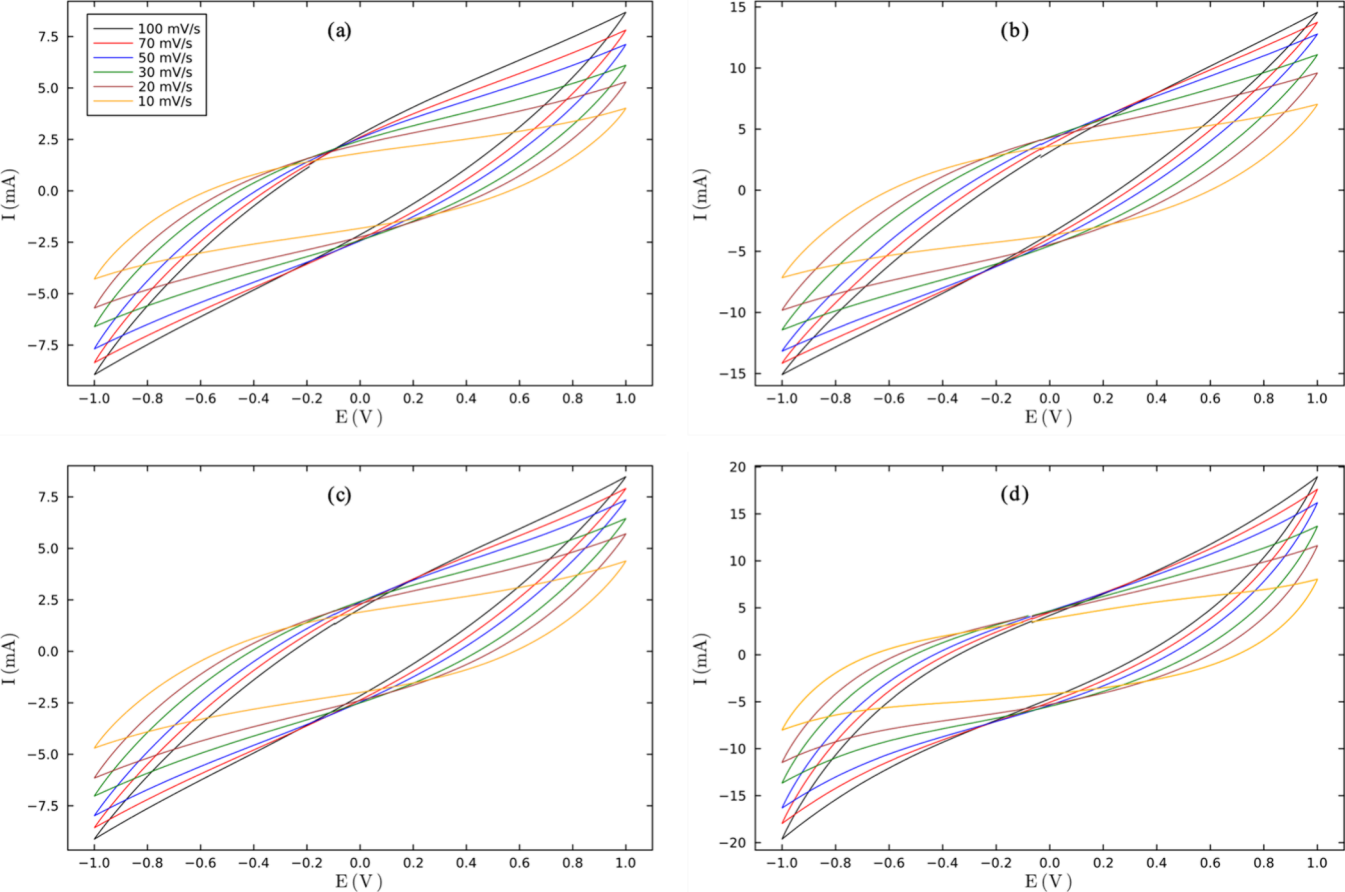
Voltammogram of SC′s for SCGPEs (a) **M1B**_**0.3**_ **T**_**0.7**_, (b) **M1B**_**0.5**_ **T**_**0.5**_, (c) **M1B**_**0.7**_ **T** **0.3**, and (d) GPE PVA-KOH.

Specific capacitance was also determined using eq 3 from the GCD profiles at different current densities of 3 to 6 mA cm^–2^, as shown in Figure 8. The capacitances obtained at 3 mA cm^–2^ were 68.16, 123.15, and 89.36 mF cm^–2^ for SCGPEs **M1B**_**0.3**_ **T**_**0.7**_, **M1B**_**0.5**_ **T**_**0.5**_, and **M1B**_**0.7**_ **T**_**0.3**_ in TFE, respectively. Similar to the cyclic voltammetry results, a higher degree of functionalization leads to greater capacitance, due to the presence of a larger number of cationic sites, which reduces the solution’s viscosity and increases charge mobility within it. The specific capacitances of both CV and GCD of the TFE-GPE devices are summarized in Table 3. This means that if the device has freer Br^–^ counterions (due to higher functionalization), more ions are available to adsorb on the carbon surface, increasing the double-layer capacitance. In the case of ionic mobility, **M1B**_**0.3**_ **T**_**0.7**_ and **M1B**_**0.7**_ **T**_**0.3**_ showed lower conductivities due to a smaller number of free Br^–^ counterions.

**Figure 8:**
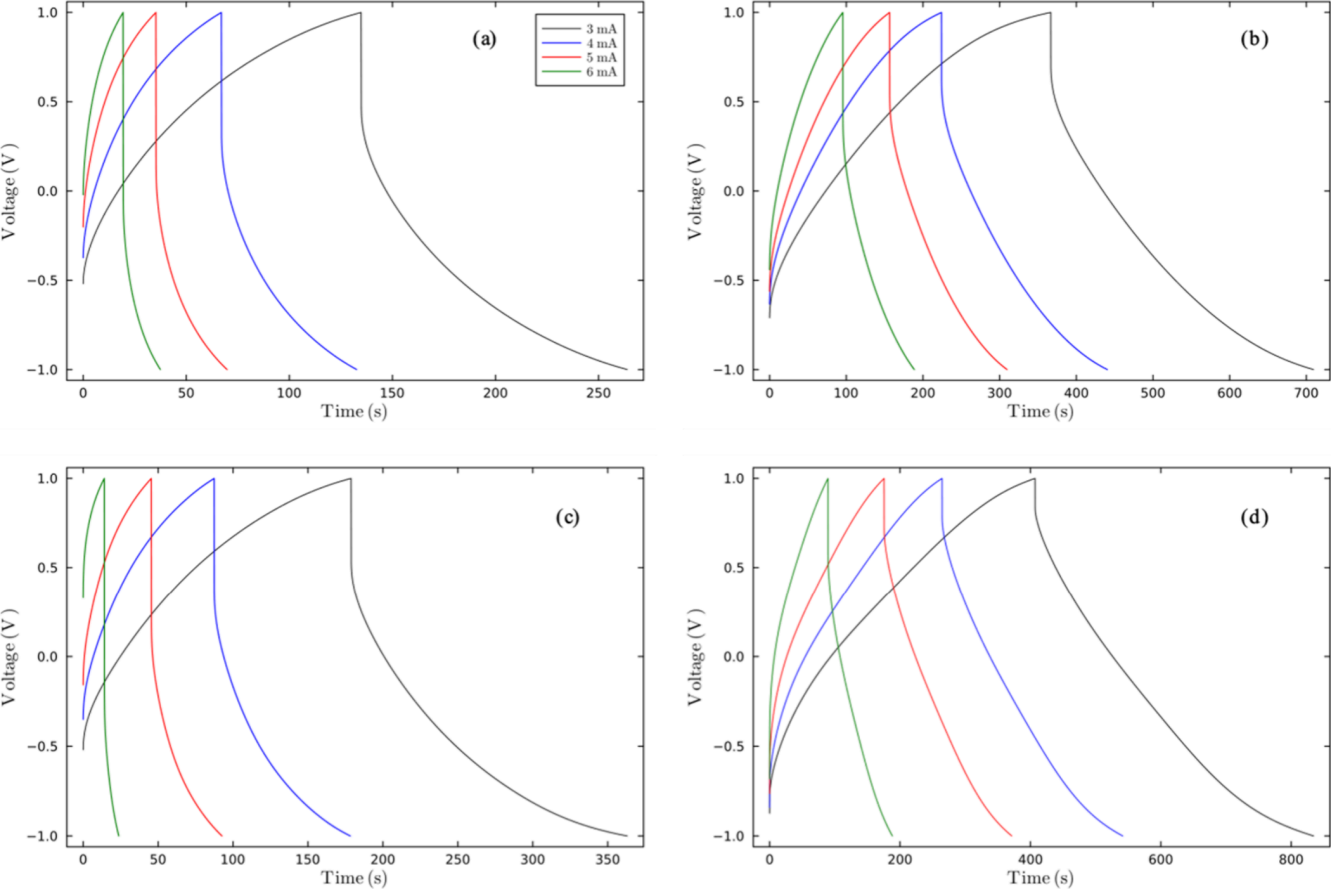
GDC profiles of SC′s for the SCGPEs (a) **M1B**_**0.3**_ **T**_**0.7**_, (b) **M1B**_**0.5**_ **T**_**0.5**_, (c) **M1B**_**0.7**_ **T** **0.3**, and (d) the PVA-KOH GPE.

Figure 9 shows the electrochemical study performed on SCs using **M1B**_**0.3**_ **T**_**0.7**_, **M1B**_**0.5**_ **T**_**0.5**_, and **M1B**_**0.7**_ **T**_**0.3**_ SCGPEs and the PVA-KOH GPE. Figure 9a shows the voltage drop (Δ*U*) obtained by the GCD curves and represents the instantaneous voltage drop at the beginning of charge or discharge steps, being a parameter that indicates ionic transport and interfacial contact resistance. A higher Δ*U* obtained by the SCs using **M1B**_**0.7**_ **T**_**0.3**_ **and M1B**_**0.3**_ **T**_**0.7**_ SCGPEs means a low ionic conductivity of the electrolyte and long ion diffusion paths. For the PVA-KOH SC, the voltage drop value is the lowest among the SCs measured, as the ions of the doped polymer are free to move. After this SC, the **M1B**_**0.5**_ **T0**_**.5**_ SC presents lower values of Δ*U*. These results are consistent with the fact that **M1B**_**0.3**_ **T**_**0.7**_ and **M1B**_**0.7**_ **T**_**0.3**_ SCGPE SCs exhibited lower capacitance and ionic conductivity as they displayed higher viscosities and lower functionalization degrees. In the same manner, another parameter related to ionic conductivity within the polyelectrolyte is the equivalent series resistance (*R*_ESR_), which is obtained from the voltage drop in GCD curves by dividing Δ*U* by the discharging current. *R*_ESR_ represents not only the solution resistance (*R*_s_), but also includes electronic and interfacial resistances that may be derived from the electrical setup used in the measurements. The same tendency with respect to ΔU is observed (Figure 9b).

**Figure 9:**
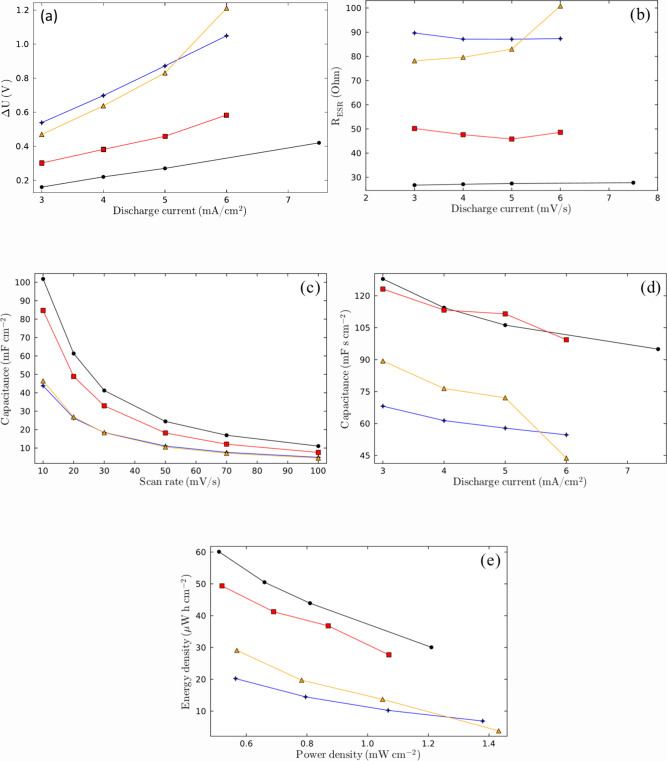
Plots of (a) drop potential, (b) resistance, (c) capacitance vs CV, (d) capacitance vs GCD and (e) Ragone plot using the (circle solid) PVA-KOH GPE and the (diamond solid) **M1B**_**0.3**_ **T**_**0.7**_, (box solid) **M1B**_**0.5**_ **T**_**0.5**_, and (triangle solid) **M1B**_**0.7**_ **T**_**0.3**_ SCGPEs.

Figure 9c,d shows the areal specific capacitances of the SCs with the aforementioned polyelectrolytes, obtained by cyclic voltammetry and GCD, respectively. Here, it can be observed that the SC with the maximum specific capacitance is observed in the PVA-KOH GPE, consistent with the free movement of KOH ions within the PVA polymer matrix. This allows greater ion movement (K^+^ and OH^–^) through the gel to the polarized carbon electrodes, resulting in a higher level of formation of EDLC sites. Here, the PVA is not considered a polyelectrolyte due to its lack of dissociated ions, and it functions only as a polymeric matrix.^11^

Among the SCGPEs, the **M1B**_**0.5**_ **T**_**0.5**_-SC showed a higher specific capacitance due to its higher functionalization degree (100%) and presented a very similar performance to that of the KOH-doped PVA GPE. Among the synthesized SCGPEs, the **M1B**_**0.5**_ **T**_**0.5**_ SC showed lower viscosity and higher ionic conductivity, allowing the movement of counterions along the polymeric chains.^22^

In **M1B**_**0.5**_ **T**_**0.5**_ SCGPE SC, the number of dissociated Br^–^ counterions is higher than in the other two SCGPEs. Here again, the SCs using the PVA-KOH SC present slightly higher performance than the **M1B**_**0.5**_ **T**_**0.5**_ GPE SC, due to their free ionic species. This same performance order is obtained in the Ragone plots, as shown in Figure 9e.

As most of the solid or gel polyelectrolytes in the literature contain an external doping agent, only a very few works correspond to truly self-charged polyelectrolytes with intrinsic conductivity.

In a very recent work, Shi et al. developed an organogel polyelectrolyte (OST/P­(AM-*co*-DMAEA-Q)/ZA) with intrinsic conductivity synthesized by a one-pot polymerization process of oxidized starch (OST), acrylamide (AM), dimethylaminoethyl acrylate methyl chloride (DMAEA-Q), and zinc acetate (ZA), and using DMSO as solvent.^31^ They reported an ionic conductivity of 1.36 mS/cm and an areal capacitance of 87.84 mF cm^–2^ when assembled in an SC device, which is slightly lower than the capacitance reported in this work, despite the ionic conductivity being higher.^31^

Although the intrinsic ionic conductivity of our higher-performance SCGPE (0.25 mS cm^–1^) is lower than that reported by Shi et al.^31^ (1.36 mS cm^–1^), the supercapacitor assembled with our SCGPE with the best performance exhibits a higher areal capacitance (120 mF cm^–2^ vs 87.84 mF cm^–2^). This result indicates that ionic conductivity alone, despite being an important parameter, does not directly determine the capacitance performance. Instead, factors such as electrolyte–electrode interface compatibility, effective ion accessibility within the porous electrode, and ion confinement at the electrode/electrolyte interface could play a dominant role in the supercapacitor performance.

## 4 Conclusions

In this work, we synthesized a dopant-free and self-charged gel-type polyelectrolyte (SCGPE) for application in textile supercapacitors. SCGPEs were obtained through a one-step superacid-catalyzed polyhydroxyalkylation of 4-acetylpyridine with biphenyl and *p*-terphenyl, followed by quaternization to introduce intrinsic positive charges into the polymer backbone. The resulting SCGPEs exhibited good solubility in polar solvent TFE and also showed high thermal stability. We found that the degree of functionalization of the SCGPE modifies both viscosity and ionic conductivity, thereby affecting the specific capacitance of an SC assembly. Electrochemical characterizations demonstrated that SCs assembled with SCGPEs exhibit ionic conductivities on the order of 10^–4^ S cm^–1^ and specific areal capacitances of up to 123 mF cm^–2^, which are comparable to those of SCs using the well-known PVA–KOH gel electrolytes. These results were obtained without the use of additional dopants, such as salts, acids, or ionic liquids. Molecular dynamics simulations further confirmed that the solvent chosen influences the ionic distribution and chain alignment when an electric potential is applied, with TFE providing the most favorable environment for charge transport. Molecular dynamics simulations confirmed that the choice of solvent governs the polymer conformation and ion mobility, supporting the superior performance observed in TFE.

## Supplementary Material



## Notes

Additional ^1^H, TGA curves, and molecular dynamics simulations of polymer fragments in TFE and DMSO under an applied electric field of 1 V (PDF)
