# Supplementary material for: Dopant-Free and Self-Charged Gel-Type Polyelectrolytes for Supercapacitors
Source: ACS Omega. 2026 Mar 17;11(12):18873–85. doi: 10.1021/acsomega.5c10696 (PMC13044830; doi:10.1021/acsomega.5c10696)
Supplement: Supplementary file 1 [file ao5c10696_si_001.pdf]

Supporting information

Dopant-free and self-charged gel type polyelectrolytes for supercapacitors

Bryan A. Corzo[1], Hugo Hernández-Martínez[1], José A. Ávila-Niño[2*], Paola G. Vilchis-Gutiérrez[3], M. Dolores Durán-García[3], Minerva Valencia-Ortega[1], Estrella Ramos[1], Lilian I. Olvera[1*]

[1]Instituto de Investigaciones en Materiales, Universidad Nacional Autónoma de México, Apartado postal 70-360, CU, Coyoacán 04510, Ciudad de México, México.

[2]Secihti - CIATEQ Unidad Jalisco, Nodo Servidor Público 165, Anexo Club de Golf Las Lomas, Zapopan, Jalisco, México, 45136

[3]Departamento de Ingeniería Mecánica, Facultad de Ingeniería, UAEMéx, Cerro de Coatepec s/n, Toluca, Estado de México, México 50110

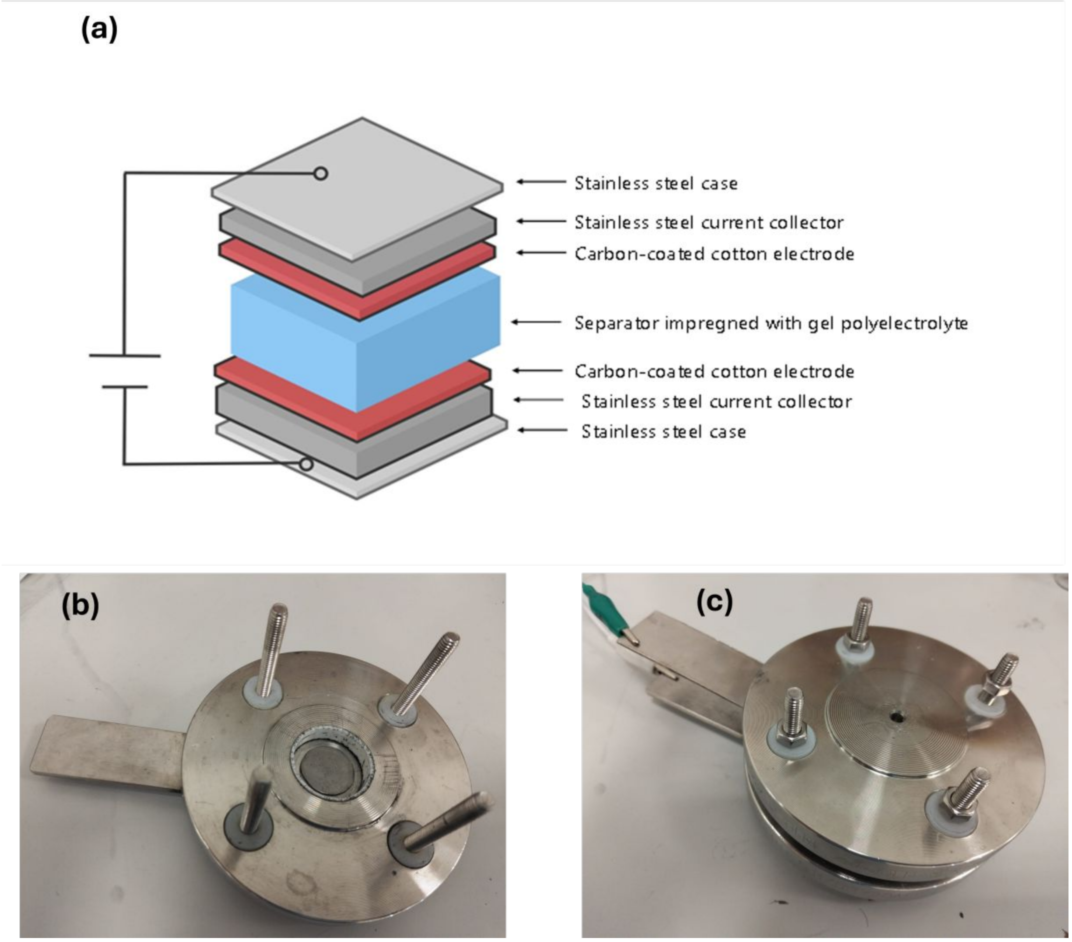

Figure S1. (a) Experimental scheme of the assembly of the 2-electrode supercapacitor device and photographs of the stainless-steel cell for electrochemical measurements (b) open and (c) closed.

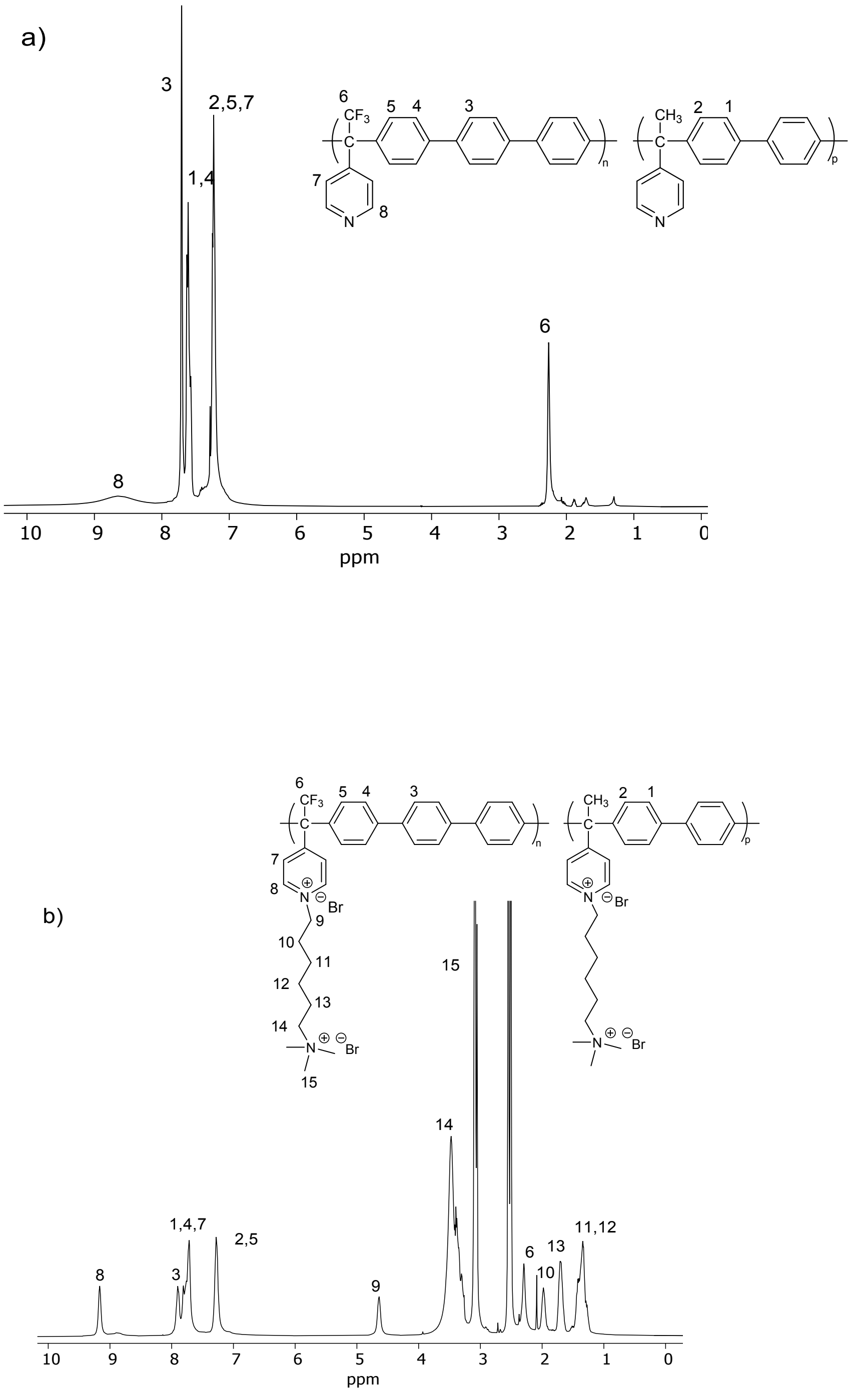

Figure S2. ^{1}H NMR spectra of (a)base copolymer $1B_{30}T_{70}$ and (b) polyelectrolyte $M1B_{30}T_{70}$.

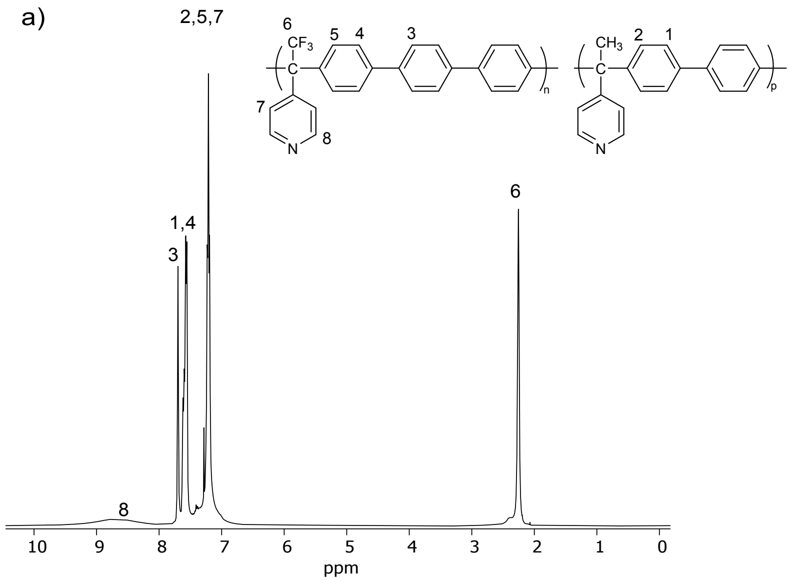

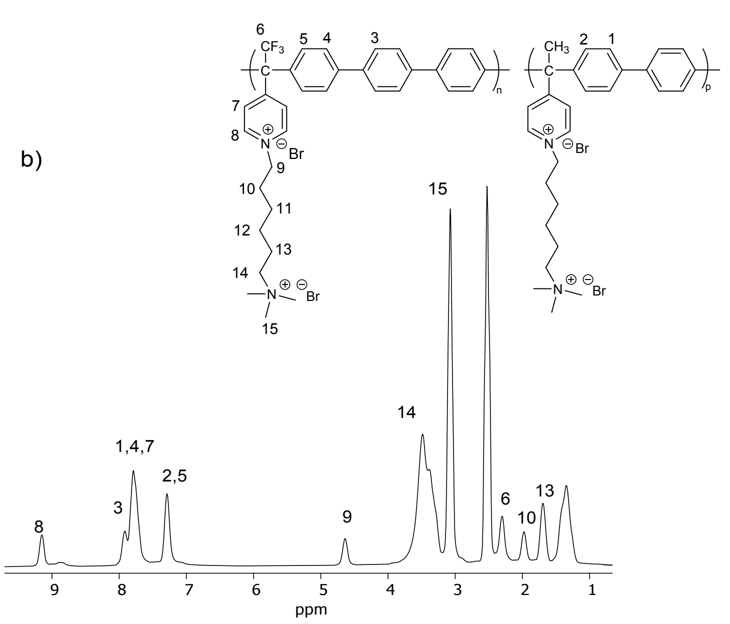

Figure S3. ^{1}H NMR spectra of (a) base copolymer $1B_{70}T_{30}$ and (b) polyelectrolyte $M1B_{70}T_{30}$.

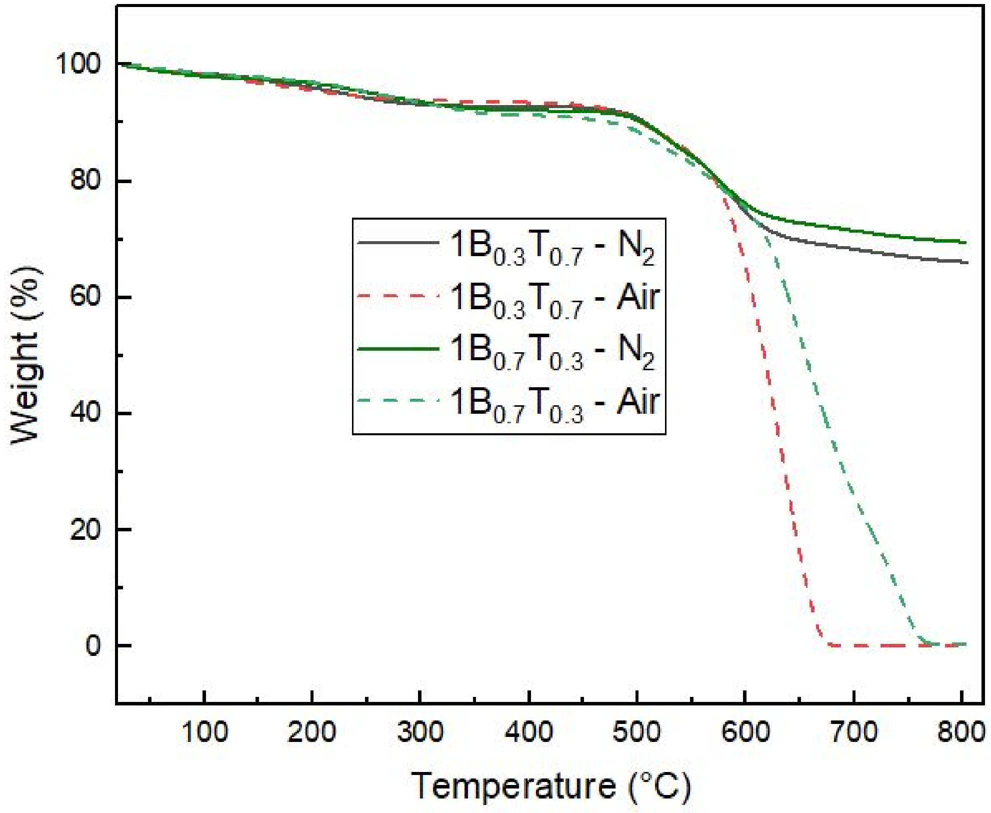

Figure S4. TGA curves $1B_{30}T_{70}$ and $1B_{70}T_{30}$ copolymers under N_2 and air atmosphere.

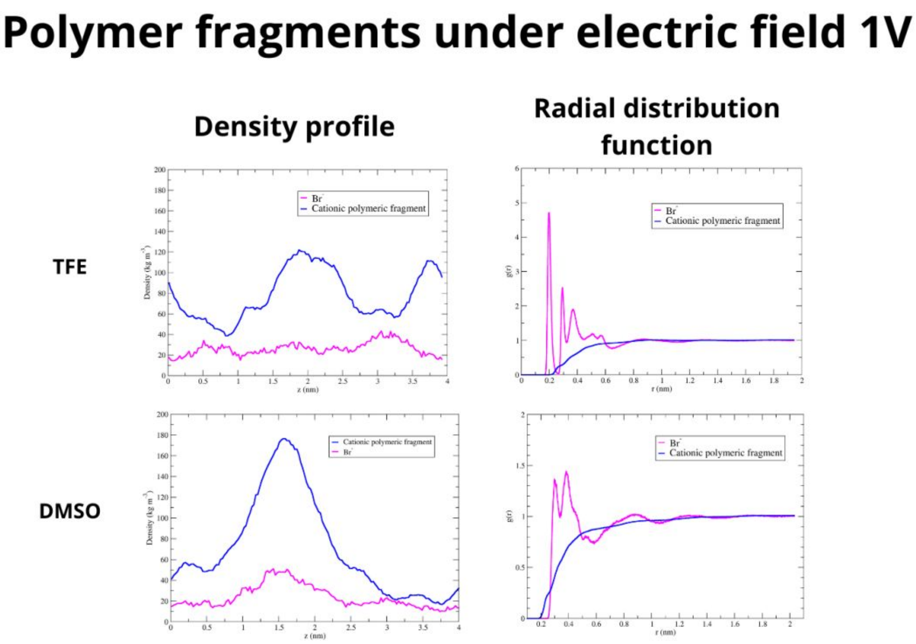

Figure S5. Molecular dynamics simulations of polymer fragments in TFE and DMSO under an applied electric field of 1 V. The density profiles are shown on the left panels, and the radial distribution functions on the right panels. The cationic polymeric fragment is shown in blue and the bromide ion in pink.

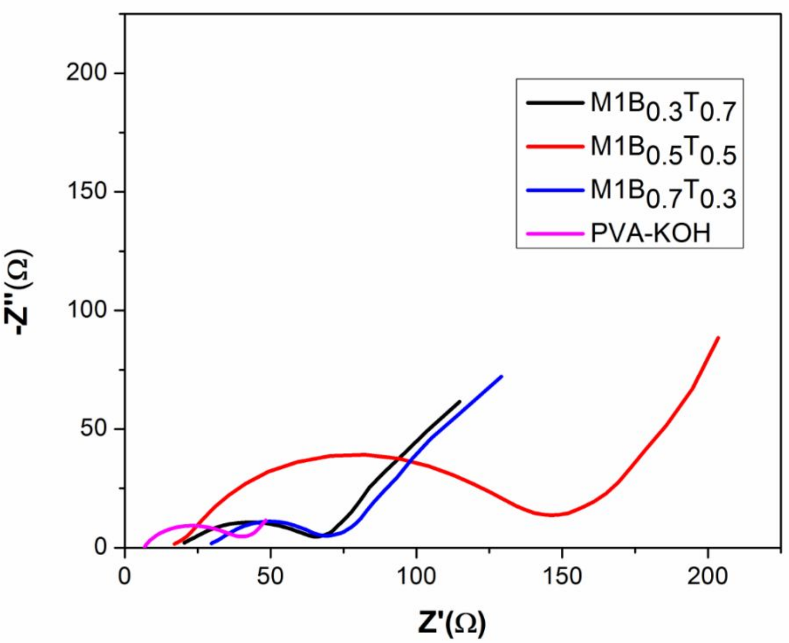

Figure S6. Nyquist plots of the SCs using the SCGPEs and the PVA-KOH polyelectrolyte.

.
